# Polycystic kidney disease complicates renal pathology in a family with Fabry disease

**DOI:** 10.1016/j.ymgmr.2022.100934

**Published:** 2022-11-14

**Authors:** Leepakshi Johar, Grace Lee, Angela Martin-Rios, Kathy Hall, Cheng Cheng, Dawn Lombardo, Madeleine Pahl, Virginia Kimonis

**Affiliations:** a Department of Pediatrics, Division of Genetics and Metabolism, University of California, Irvine, CA, USA; b College of Osteopathic Medicine of the Pacific, Western University of Health Sciences, Pomona, CA, USA; c Department of Medicine, Division of Nephrology, University of California, Irvine, CA, USA; d Division of Nephrology, University of California, Irvine, CA, USA

**Keywords:** Fabry disease, Polycystic kidney disease, PKD1

## Abstract

Fabry disease is a rare lysosomal storage disorder that primarily affects the heart and kidneys, often presenting with reduced renal function. Polycystic kidney disease is a renal condition in which cysts are found, which have a different presentation than the cysts associated with Fabry disease. We report a 60-year-old male patient who was diagnosed with Fabry disease with the classic c.730G > A (p.Asp244Asn) variant of the *GLA* gene at 34 years of age. Fabry symptoms in this patient include hypohidrosis, hearing loss, corneal whorling, and edema. He also presented with polycystic kidney disease with multiple simple and mildly complex cysts on abdominal ultrasound. Family history of note included Fabry disease in his mother and maternal uncle as well as polycystic kidneys in his mother. Molecular analysis for polycystic kidney disease revealed a variant of uncertain significance (VUS) in the *PKD1* gene. Although the in silico studies of this VUS have inconclusive results, the patient fills clinical criteria of autosomal dominant polycystic kidney disease, therefore, Fabry disease and polycystic kidney disease are considered two co-existing manifestations in this family. This case demonstrates the possibility of two renal comorbidities in the same individual and the risk of one diagnosis being overlooked by the other.

## 1 Introduction

Fabry disease is a rare X-linked lysosomal storage disorder caused by pathogenic variants in the *GLA* gene, which encodes for the α-galactosidase A enzyme [1]. This results in the absence or remarkably low activity of this enzyme which leads to reduced ability to metabolize glycosphingolipids and consequent accumulation of globotriaosylceramides (GL3 or Gb3) [2]. The accumulation of globotriaosylceramides lead to cell and organ dysfunction, affecting many organ systems including the blood vessels, heart, kidneys, skin, nervous system, and the brain [2].

The multi-organ clinical manifestations of Fabry disease include but are not limited to corneal opacities, angiokeratomas, anhidrosis, acroparesthesias, abdominal pain, constipation, and diarrhea. Fabry-related renal assessments include proteinuria and progressive kidney damage that may lead to dialysis or renal transplant [1]. Cardiac findings include arrhythmias, left ventricular hypertrophy, and hypertrophic cardiomyopathy. Currently, approved therapies for Fabry disease include α-galactosidase enzyme replacement therapy (ERT) and a protein chaperone which increases α-galactosidase A enzymatic activity [3,4].

Polycystic kidney disease (PKD) is an autosomal dominant or autosomal recessive inherited kidney disorder associated with multiple clusters of cyst formation [5]. Autosomal recessive polycystic kidney disease (ARPKD) is associated with variants in the *PKHD1* gene with manifestation typically in infancy or in childhood [6]. Given the early and sometimes severe manifestations of this condition, management is supportive [7]. Autosomal dominant PKD (ADPKD), which more commonly is caused by variants in the *PKD1* or *PKD2* gene, manifests between 30 and 50 years of age [6]. PKD is associated with flank pain, hematuria, hypertension, kidney stones, end-stage renal disease, liver/pancreatic cysts, and brain aneurysms [5]. Initial treatment for ADPKD consists of management of blood pressure, dietary sodium restriction and increased fluid management [5]. Patients at high risk for progression are offered treatment with tolvaptan [8]. We report a patient and family with Fabry disease who presented with renal cysts found to be associated with autosomal dominant polycystic kidney disease [1].

## 2 Case report

We report a 60-year-old male patient who was diagnosed with Fabry disease at the age of 34 years with the classic c.730G > A (p.Asp244Asn) variant of the *GLA* gene and treated with agalsidase beta biweekly. The patient reported fatigue, hypohidrosis, hearing loss, corneal whorling, and mild edema in the extremities. Past medical history consisted of a diagnosis of ulcerative colitis at 42 years of age for which he was on mesalamine with remission of the disease. He also had history of recurrent diarrhea secondary to Fabry disease. He was followed by cardiology due to high blood pressure and mild to moderate aortic root dilation detected through echocardiogram at 58 years of age. Hypertension was managed with losartan 100 mg daily. His physical exam revealed a blood pressure of 127/72 mmHg with a pulse of 65 bpm. He had few angiokeratomas in the extremities, no abdominal masses, and no other clinical findings. Relevant laboratory findings included: sodium of 140 mEq/l, potassium 4.9 mEq/l, bicarbonate of 26 mEq/l, and a creatinine of 1.24 mg/dl. Serum creatinine level eight years before was noted to be 1.1 mg/dl and his most recent creatinine level was 1.24 mg/dl. Urine analysis revealed a specific gravity of 1.010, negative Dipstick evaluation for protein and hemoglobin and normal a microscopic examination. Urinary microalbumin to creatinine ratio was 152 mg/mg 8 years prior; a current value, on losartan, was 47 mg/mg. His serum cholesterol was 175 mg/dl with an HDL 55 mg/dl and an LDL of 101 mg/dl. His most recent echocardiogram performed at age 59 years showed mild left atrial enlargement, mild mitral annular calcification, mild mitral regurgitation, and moderately dilated aortic root, with no significant change from his previous echocardiogram two years earlier. The patient routinely follows up with his cardiologist and nephrologist as part of his care. Routine ultrasound at the time of diagnosis identified multiple cysts in the kidneys.

**Fig. 1:**
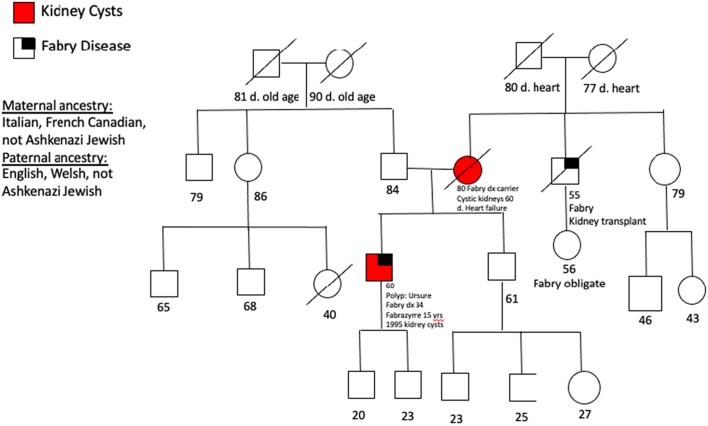
Family pedigree of the patient with pertinent family history. The patient and his mother had renal ultrasounds that showed multiple bilateral cysts. Males are depicted by squares, females are circles, deceased pts. indicated by a slash.

Relevant family history (Fig. 1) includes a mother with Fabry disease diagnosed at the age of 60 years who had shortness of breath, congestive cardiac failure, hearing loss, and multiple bilateral renal cysts, treated with ERT upon diagnosis. She passed from heart failure at the age of 80 years. His maternal uncle was also affected with Fabry disease and received long-term dialysis and multiple kidney transplants. He ultimately passed away due to renal failure at the age of 55 years. It is unknown whether the maternal uncle had renal cysts. Renal ultrasounds were not available on other first degree relatives. Family history of hypertension was reported on both paternal and maternal sides of the family. His maternal ethnic background is Italian and French-Canadian descent, and his paternal ethnic background is of English and Welsh descent.

**Fig. 2:**
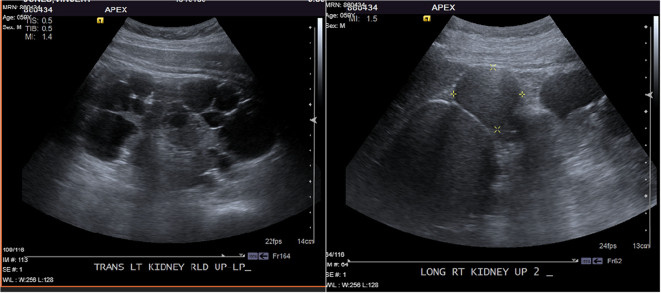
Abdominal ultrasound images of renal cysts in patient. Multiple cysts were noted in the left kidney and some of which were complex. The largest and most complex one measured 7.3 cm. The largest cysts in the right kidney were located in the lower pole with a size of 4.2 cm.

An abdominal ultrasound at age 57 years showed the right kidney measuring 20.2 cm and the left measuring 16.7 cm in length. The right kidney had multiple large cysts with internal echogenicity. These cysts were categorized as are most likely hemorrhagic or proteinaceous. The largest lesions were in the upper pole of the right kidney that contain internal echogenicity and measured 8.1 × 8.0 cm and 5.1 × 4.9 cm. In the left kidney, there were multiple benign simple and complex cysts, measuring up to 5.6 cm in diameter. The sizes of the cysts had increased from prior ultrasounds done 1–3 years earlier. No definite solid lesion of the kidneys was reported (Fig. 2).

Abdominal ultrasound at age 57 years identified an echogenic lesion in the right hepatic lobe with a size of 1.1 cm. An echogenic lesion in the right hepatic lobe measured 1.2 × 1.1 × 1.2 cm. The cyst in the left hepatic lobe measured 1.0 × 0.7 × 1.0 cm. There was also a small cyst in the right hepatic lobe, measuring 0.1 cm. The small cyst in the right lobe and the cyst in the left lobe increased in size from prior ultrasounds done 1–3 years earlier.

Due to the finding of multiple cysts in his kidneys, the patient was referred for genetic testing and underwent testing of genes associated with polycystic kidneys: *GANAB*, *HNK1B*, *PKD1*, *PKD2*, *PKHD*, and *PRKCSH.* He was identified a c.11713-5C > A intronic variant considered a variant of unknown significance (VUS) in the *PKD1* gene. In-silico analysis that included splice site predictors and regions of evolutionary conservation was inconclusive regarding whether this mutation results in alternative gene splicing and is classified as a variant of unknown significance (VUS). At risk family members including his brother, father, and sons were advised to undergo *PKD1* familial variant genetic testing, and kidney imaging such as ultrasound or MRI to assess for renal cysts, however this was not pursued.

## 3 Discussion

We report a patient with Fabry with the classic c.730G > A (p.Asp244Asn) variant of the *GLA* gene and large complex renal cysts identified as *co*-existing polycystic kidney disease. According to the Pei-Ravine criteria [9], the presence of at least three renal cyst and two cysts in each kidney are diagnostic for ADPKD in individuals at risk with unknown genotype. The finding of multiple bilateral cysts in our 57-year-old patient confirms the clinical diagnosis of ADPKD. The association of kidney cyst with liver cyst and aortic root dilation also supports this diagnosis [6].

Based on the patient's genetic results for PKD, the c.11713-5C > A variant was found in intron 42 of the *PKD1* gene. This variant is located in the region of the gene that typically tolerates mutations. In-silico analysis that included splice site predictors and regions of evolutionary conservation was inconclusive regarding whether this mutation results in alternative gene splicing. However, four *PKD1* variants have been reported around this intronic region in the ClinVar database, including c. 11713-1G > A, c. 11713-2A > G, c. 11713-3C > T and c. 11713-3C > G. Variants *PKD1* c. 11713-1G > A and c. 11713-3C > T were reported to be associated with ADPKD [10]. To compare our novel variant with the above-mentioned variants and evaluate their pathogenicity, we utilized the regSNP-intron software to predict pathogenic impact of the intronic single nucleotide variants [11]. Importantly, *PKD1* c. 11713-1G > A, previously identified as a pathogenic variant in a germline-mutation screening studies on individuals with a family history of ADPKD [10], is highly conserved across species and was predicted to be damaging by regSNP-intron software. In addition, both *PKD1* c. 11713-2A > G and c. 11713-3C > G variants were predicted to be damaging. However, our novel variant, c.11713-5C > A, along with variant c. 11713-3C > T were predicted to be benign despite both being associated with ADPKD. The nucleotide at the c.11713-5C position is not conserved across evolution. Nevertheless, we lack functional studies to assess whether the novel variant will disrupt normal RNA-splicing events— thus, its effect remains unknown and was classified as a variant of unknown significance (VUS). Based on the Lek et al. study [12], this variant has been observed in 0.0092% of large population cohorts– 8 alleles among the 87,046 alleles that were analyzed. Since the location of the variant is adjacent to known pathogenic variants, and the clinical features of the patient (presence of >2 cysts in each kidney) plenty supports the diagnosis of ADPKD, we believe the VUS should be reclassified as a pathogenic variant.

Reports of renal cysts associated with Fabry Disease in literature are scarce. The cysts in Fabry disease are typically parapelvic and smaller than those described in our patient. In a nationwide cohort of 173 patients evaluated for renal prevalence via ultrasound, parapelvic cysts were detected in 28.9% of the cohort as compared to 1.1% (*p* < 0.001) in the control group [13]. The size of these cysts ranged from a few millimeters to 8 cm. In their second study [14], 67 participants from a single center were evaluated for parapelvic cysts to determine whether parapelvic-cyst detection in Fabry disease is more accurately determined using this method. Based on the results, there was a 43.3% parapelvic cyst prevalence (*p* < 0.05). Pisani et al.(2018) concluded that patients with parapelvic cysts and an unclear history of renal disease should raise the suspicion for Fabry Disease [13].

Ries et al. (2004) evaluated 24 patients with chronic neuropathic pain that participated in an ERT trial in a cross-sectional case-control study; they found that 50% of the Fabry disease patients had renal sinus/parapelvic cysts detected by renal MRI and CT imaging, as compared to 7% in a control group of healthy males [15]. Glass et al. (2004) [16] Found that among the 76 affected males, 64.5% had renal abnormalities, including cysts, decreased cortical thickness, and loss of corticomedullary differentiation. Among 40 females, 60% had renal abnormalities that were detected in ultrasound and/or MRI scans including parapelvic cyst. Our patient's cyst were not parapelvic and were larger than those noted in prior studies.

A review of the literature identified another case with similar findings to our patient: a 54-year-old male with multiple renal cysts and Fabry disease (*GLA* g.10571C > A mutation) [14]. The patient had a prior history of mild articular pain and hypohidrosis, and at the age of 39 years developed a myocardial infarction, followed by angina and multiple and transient ischemic strokes until 48 years of age. The abdominal ultrasound and T2 weighted MRI revealed multiple bilateral renal cysts and hepatic cysts. He had normal renal function. There was no family history of PKD. Fabry disease and ADPKD were thought to be separate disorders in this patient, similar to our patient [14].

Given the rarity of Fabry disease, it is common to misdiagnose the manifestations of this disease. However, the finding of cyst that fills the Pei-Ravine criteria [9] led us to investigate an additional diagnosis in a patient: ADPKD. A thorough clinical evaluation and the careful review of tests of any patient with discordant findings should raise the suspicion of a second diagnosis as noted in this patient.—.

## Declaration of Competing Interest

None.

## Data Availability

Data will be made available on request.
